# Gastrocsoleus lengthening in combination with tibialis anterior tendon shortening for equinus deformity in children with cerebral palsy: a systematic review

**DOI:** 10.1186/s13643-025-02985-y

**Published:** 2025-11-20

**Authors:** Devam Modi, Daniel Gould, Ken Ye, Kerr Graham, Erich Rutz

**Affiliations:** 1 Department of Orthopaedics, The Royal Children’s Hospital, Melbourne, 3052 Australia; 2 Department of Paediatrics, University of Melbourne, Parkville, VIC 3052 Australia; 3 Murdoch Children’s Research Institute, Melbourne, 3052 Australia; 4 Department of Paediatrics, Bob Dickens Chair, Paediatric Orthopaedic Surgery, The University of Melbourne, Melbourne, 3010 Australia; 5 Medical Faculty, University of Basel, Basel, 4001 Switzerland

## Abstract

**Background:**

Equinus is the most common deformity in cerebral palsy, and gastrocsoleus lengthening (GSL) is the most common surgical intervention in children with cerebral palsy (CwCP). GSL is a dose-dependent surgical concept carried out with consideration of the severity of equinus contracture, aimed at addressing this problem during the stance phase of gait. The addition of the novel procedure, tibialis anterior tendon shortening (TATS), may offer benefit in correcting the swing phase problem by addressing foot drop due to the agonist/antagonist relationship with gastrocsoleus. Given the small sample sizes and mixed results presented by current literature, our study aimed to collate the available evidence in order to review the effectiveness and safety of the addition of TATS to the GSL procedure for CwCP with equinus deformity.

**Methods:**

In this PRISMA (Preferred Reporting Items for Systematic reviews and Meta-Analyses)-compliant systematic review, a systematic search of Medline, Embase, Web of Science, and Google Scholar retrieved 674 articles for title and abstract screening. Five original publications were included in the final review. The methodological index for non-randomised studies (MINORS) was used to critically appraise included studies.

**Results:**

Sample sizes ranged from 20 to 29, with a mean age at surgery ranging from 10.0 to 16.8 years. All participants underwent pre- and post-operative 3D gait analysis. Variations regarding the technique of the procedure included the suture material used to complete TATS and the anatomical level/zone of GSL. Studies found that combined GSL in combination with TATS demonstrated an improvement in gait profile score (GPS), Gait Variable Score (GVS) at the ankle, and reduced ankle–foot orthosis (AFO) use. No long-term complications were detected because of adding TATS to GSL.

**Conclusion:**

The GSL in combined with the TATS procedure appears to be a safe procedure with a low complication rate, which may offer improvements in GPS, GVS, and reduced AFO use; however, comparative studies will assist in identifying the effect this has beyond GSL only and the patients for whom this procedure will provide the best outcomes.

**Systematic review registration:**

Open Science Framework (https://osf.io/dp7ha/)

**Supplementary Information:**

The online version contains supplementary material available at 10.1186/s13643-025-02985-y.

## Introduction

Equinus is the most common deformity in cerebral palsy and results in the inability to dorsiflex the foot above plantigrade [1]. A recent Delphi consensus outlined that the aims of management of equinus in cerebral palsy were prevention of deformity, correction of deformity, promotion of a base of support, facilitation of training skills, and an improvement in gait efficiency [2]. Equinus is initially managed with casting and Ankle foot orthoses (AFOs), designed to allow dorsiflexion but limit plantar flexion. Botulinum toxin may also be indicated in early treatment of equinus deformity [3]; however, as the disease progresses and gastrocsoleus contracture develops, surgery is indicated [4]. Most procedures involve lengthening the gastrocsoleus (GSL); however, variations exist [2]. Zone one surgery is usually performed close to the termination of the medial gastrocnemius muscle belly and the region where the gastrocnemius aponeurosis and soleus fascia become conjoined. This permits a choice between lengthening only the gastrocnemius aponeurosis and lengthening both the gastrocnemius aponeurosis and soleus fascia. Zone two GSL is performed at the level of the conjoined gastrocsoleus tendon. Zone three GSL is the lengthening of the achilles tendon. The current consensus is to perform a zone one GSL in children with diplegia and zone two or three surgery for children with hemiplegia [2]. Despite a technically satisfactory GSL and successful correction of the stance phase gait problem, foot drop may persist, especially in children with hemiplegia [5]. Foot drop is perceived as a limp and may reduce satisfaction with the outcome of GSL surgery and require continued use of an AFO [2].

In 2011, Rutz [6] described a novel surgical procedure, tibialis anterior tendon shortening (TATS), for comprehensive management of equinus in children with cerebral palsy (CwCP), in which surgery is directed at both the stance phase problem (equinus) and the swing phase problem (foot drop). Dose-based GSL is required for management of equinus deformity and gait; then, TATS is added for management of foot drop in the swing phase. This combined approach (GSL and TATS) showed improvements in both the stance phase and swing phase [6]. These findings have been corroborated by three studies [7–9]; however, Dussa [10] published conflicting results.

Given the mixed results and small sample sizes of the published literature, the aim of this review was to synthesise the current evidence regarding GSL in combination with TATS for the management of equinus deformity in CwCP. Considering the limited literature present regarding this novel procedure, a systematic review was conducted to synthesise the available evidence and lay the foundation for prospective studies to address specific outstanding research questions pertaining to this complex topic. Previously published reviews on novel surgical procedures or in patients with relatively uncommon conditions often incorporate case series and studies with small sample sizes, while also commonly comprising a small number of studies [11–15]. This informed the review team’s rationale for conducting the current review, while also guiding study design and manuscript preparation.

## Methods

A systematic review was conducted in accordance with the PRISMA (Preferred Reporting Items for Systematic reviews and Meta-Analysis) guidelines [16]. The study protocol was prospectively registered with Open Science Framework on the 15th of July, 2024 [17].

### Search strategy

On July 6th 2024, an electronic search was performed in line with prior literature on optimal search strategy development [18]. This included MEDLINE, EMBASE, Web of Science, and Google Scholar, all searched from inception. Keywords used in the search strategy included “Cerebral palsy”, “Equinus”, “Tendons”, “Tibialis anterior”, “Gastrocsoleus” and “Surgery”. Complete search strategies are included in the supplementary file. Forward and backward citation searching was conducted for all included articles. All search results were then imported into Covidence [19] for screening.

### Eligibility criteria

Studies were included if they pertained to the management of equinus in CwCP, included GSL (of any type) and TATS in one procedure, with outcomes assessed by three-dimensional gait analysis (3DGA) with a minimum 1 year follow-up. Studies which investigated other surgical procedures or non-cerebral palsy populations were excluded. Eligibility criteria were clarified through discussion between reviewers DM and DG following screening of 10% of retrieved titles and abstracts, overseen by senior author ER.

### Data extraction

The included studies were examined, and data were extracted using a custom form. This form was developed with input from senior author ER and improved through pilot testing and feedback. It focused on study sample characteristics, surgical intervention, and outcome measures. The full data collection form can be found in the supplementary file.

### Critical appraisal

Risk of bias assessment was completed using the Methodological index for non-randomised studies (MINORS) [20]. This critical appraisal tool is used to evaluate studies through eight domains, each assigned a score from zero to two. Scores are combined to develop a final risk of bias rating for each study, with lower results indicating a higher risk of bias.

### Data synthesis

Results from included studies were deemed unsuitable for quantitative synthesis through meta-analysis due to a high degree of sample dependence, variability in data reporting, and small sample size. Narrative synthesis was conducted, with data presented graphically and in summary tables, in concordance with Synthesis without Meta-analysis (SWiM) guidelines [21]. This review utilised the Grades of Recommendation, Assessment, Development, and Evaluation (GRADE) [22] system for the synthesis of results.

## Results

### Search strategy yield

**Fig. 1:**
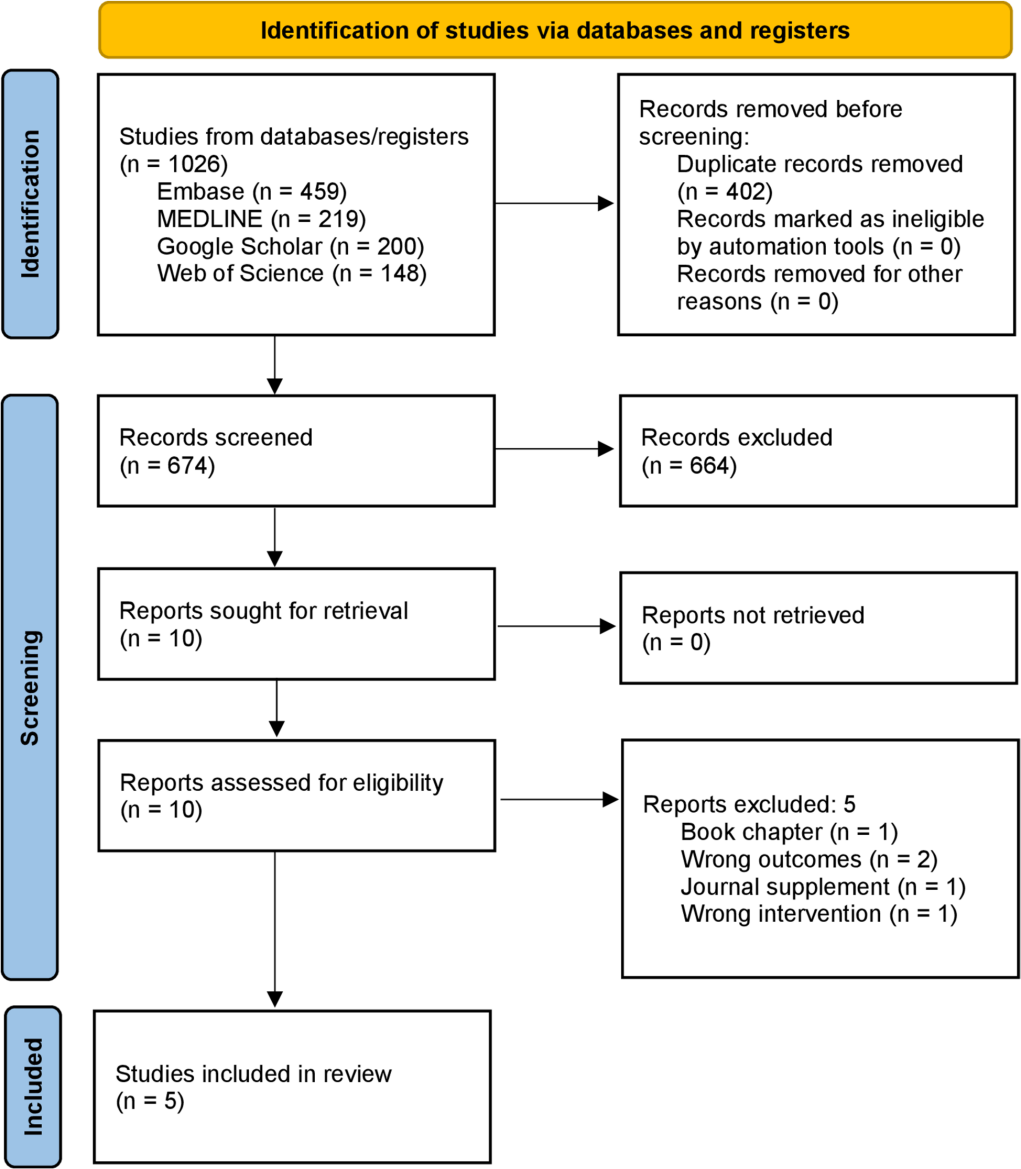
PRISMA diagram

Out of 674 articles screened, 664 were excluded. The full texts of 10 studies were reviewed, of which two reported unrelated outcomes, one was a book chapter, one was a conference abstract, and one studied the wrong intervention. This left five studies for narrative synthesis. Figure 1 shows the PRISMA diagram summarizing these results.

Table 1 contains a summary of the descriptive aspects of included studies.

**Table 1:** Descriptive aspects of studies

Author	TATS procedure	GSL	SEMLS	Groups	No. of subjects	No. of operated limbs	Age (years) Mean ± SD (range)^a^	GMFCS	Outcome measures	Follow-up (months) Mean ± SD (range)^a^	MINORS score (scored out of 16)
Rutz et al. (2011) [6]	“Rutz procedure”	Zone 3	N/A	Hemiplegic	21	21	15.1 ± 6.3 (7.0–37.5)	I: 9 II: 20	Clinical exam, 3DGA, EMG, Force plates	14.0 (9.0–24.0)	9
				Diplegic	4	8				14.3 (9.0–24.0)	
Tsang et al. (2016) [7]	“Rutz procedure”	Zone 1	Asynchronus MLS	Hemiplegic	13	13	16.8 ± 5.9 (10.3–34.5)	I: 18 II: 8	Clinical exam, 3DGA	17.1 ± 5.6	12
				Diplegic	13	15					
Kläusler et al. (2017) [8]	“Rutz procedure”	Zone 3	14 single procedure, 5 SEMLS	Hemiplegic	12	12	13.3 ± 3.0	I: 7 II: 12	Clinical exam, 3DGA, EMG, Force plates	69.6 ± 25.2	8
				Diplegic	8	10	16.6 ± 5.1	I: 1 II: 7			
Dussa et al. (2021) [10]	“Rutz procedure” with modification	Zone 1 or 3	"Many had SEMLS"	Hemiplegic	12	12	13.5 (6.0–19.0)	I/II	Clinical exam, 3DGA, Force plates	19.7 ± 3.2	8
				Diplegic	9	9	10.0 (5.0,34.0)				
Widmer et al. (2024) [9]	“Rutz procedure” with modification	Zone 3	N/A	Hemiplegic	22	22	13.3 ± 3.0	I: 18 II: 4	Clinical exam, 3DGA, Force plates	N/A	9

^a^Standard deviation and range included where possible from data available from original studies

Study sample sizes ranged from 20 to 29 subjects. In some studies, it was difficult to identify the precise sample size given that diplegic subjects may have been managed with unilateral or bilateral surgery. In these cases of ambiguity, sample sizes were estimated based on the data provided.

Mean ages of study participants ranged from 10.0 to 16.8 years. Pre-operative Gross Motor Function Classification System (GMFCS) levels [23] ranged from I to II. In studies which included two cohorts, group one comprised children with hemiplegia, and group two comprised children with diplegia. Notably, all children were ambulatory pre and post-operatively.

Three of five included studies, Rutz [6], Kläusler [8], and Widmer [9], were conducted at the same institution. They included overlapping study periods and investigated subjects with similar inclusion criteria. It is plausible that some participants were included in more than one study. It was not possible to identify and remove data for subjects included in multiple studies. This resulted in sample dependence. Accounting for this, Kläusler [8] was selected as the representative study given it comprises long-term follow-up of the subject cohort included in Rutz [6] and, unlike Widmer [9], it did not exclude subjects who had bilateral surgery.

### Operative intervention

Surgical methods for TATS were consistent across Rutz [6], Tsang [7], and Kläusler [8], using 6 Vicryl (Ethicon Inc., Johnson and Johnson) for the tendon suture. However, Dussa [10] elected to use a 1.0 Polydioxanone suture (PDS Ethicon®), and Widmer [9] used a 2.0 FiberWire (Arthrex Manufacturing Inc.) from 2020 onwards to complete TATS. Tsang [7] and Dussa [10] chose to complete zone one GSL rather than zone three described in Rutz [6].

**Fig. 2:**
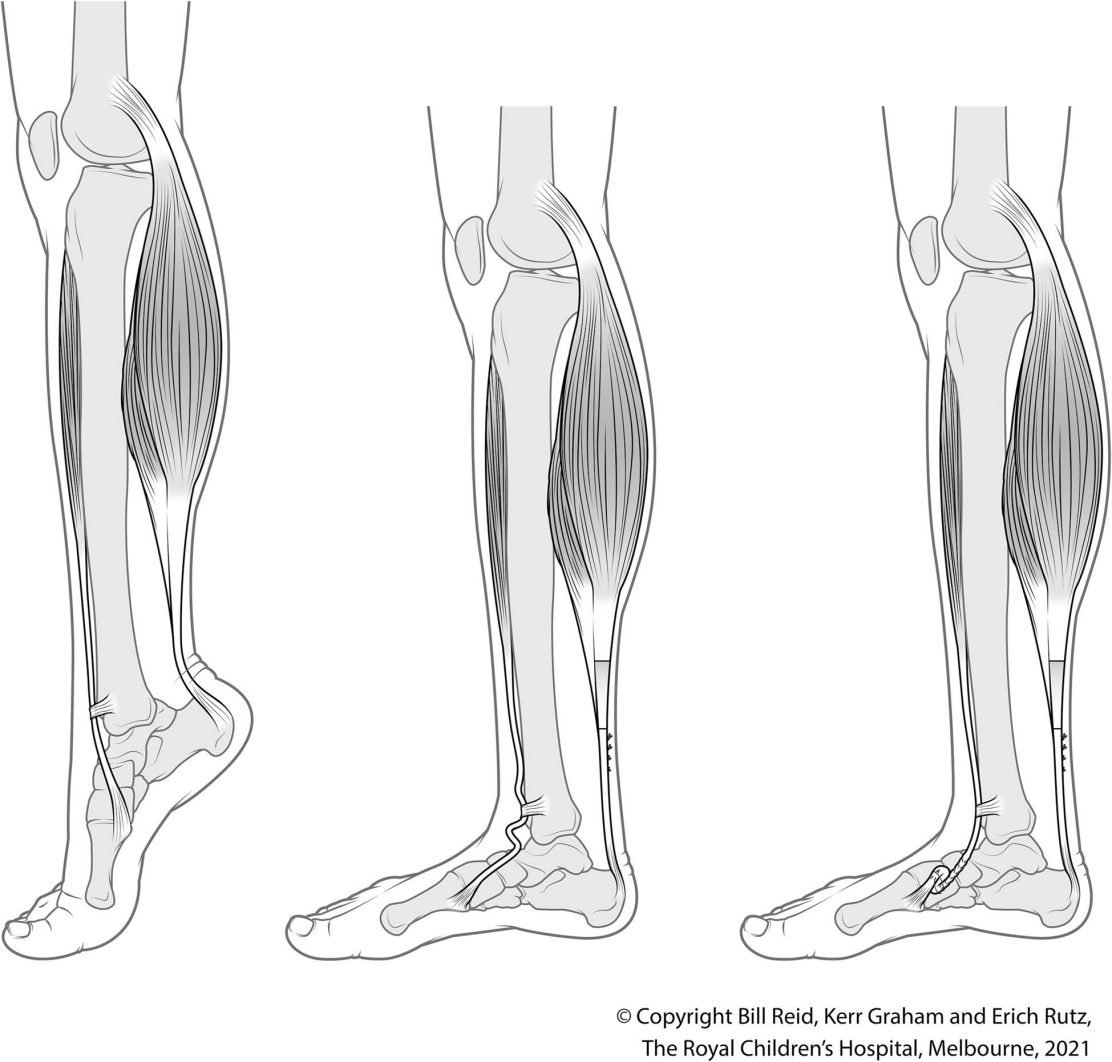
The figure depicts preoperative deformity, effect of GSL leaving a lax tibialis anterior tendon, and the effect of the combination of the GSL and TATS procedure

Figure 2 depicts preoperative deformity, the effect of GSL leaving a lax tibialis anterior tendon, and the effect of the combination of the GSL and TATS procedure.

**Fig. 3:**
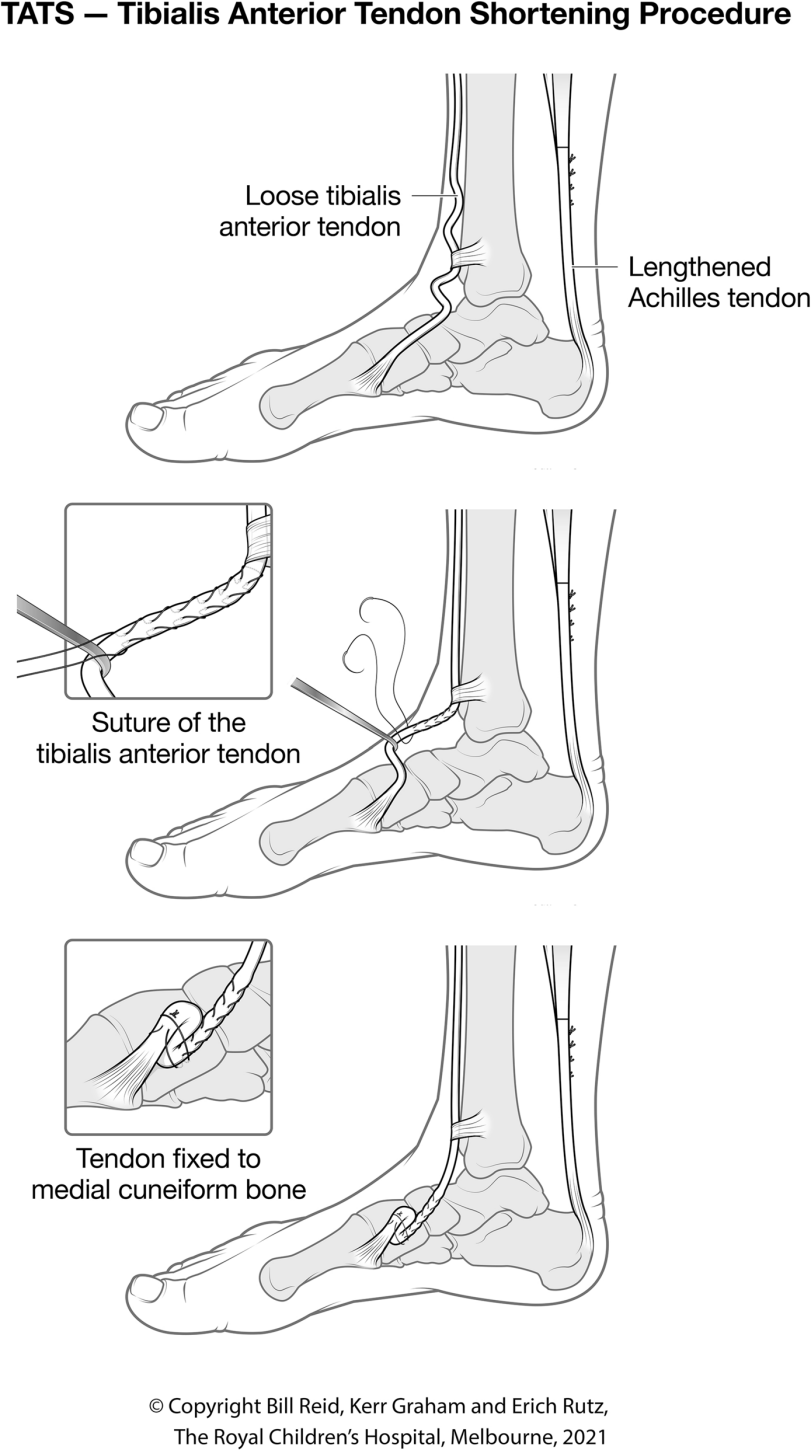
Technique of shortening the tibialis anterior tendon and securing the shortened tendon to the medial cuneiform with transosseous fixation

Figure 3 depicts the technique of shortening the tibialis anterior tendon and securing the shortened tendon to the medial cuneiform with transosseous fixation.

### Post-operative care

Post-operatively, subjects were placed in a plantigrade below-knee plaster cast, with weight-bearing commencing 2 to 6 weeks after surgery. After 6 weeks, the cast was either removed completely or replaced with an AFO.

### Outcome assessment

All participants were reviewed pre-operatively and received their first post-operative evaluation between six and 24 months after surgery. Kläusler [8] studied long-term outcomes for Rutz [6] study cohort, with post-operative follow-up duration of 5.8 years (± 2.1 years standard deviation (SD)).

Outcomes assessed included clinical examination, temporospatial measures, kinematics, muscle spasticity, muscle strength, and gait scores. All studies included instrumented 3D gait analysis using a Vicon camera system (Oxford Metrics Ltd., UK). Additionally, some studies used force plates (Kistler instrumente AG, Winterhur, Switzerland) and surface electromyography (EMG) system (Neurodata GmBH, Vienna, Austria). This consistency in data collection methods allowed for comparison in the included studies.

### Quality of evidence

All studies included for review were of level four evidence according to Oxford Centre for Evidence-Based Medicine (CEBM) criteria levels of evidence. MINORS [20] scores ranged from eight to twelve out of sixteen, showing methodological heterogeneity between included studies. A summary of these findings is included in Table 1 and in full form in supplementary materials. As all included studies were case series, evidence from these would be rated as “very low quality” as per GRADE criteria [22].

### Gait Profile Score (GPS)

Rutz (hemiplegia (*n* = 21): pre-op 12.47 ± 3.80 to post-op 9.18 ± 2.55 (*p* = 0.0022), diplegia/quadriplegia (*n* = 8): pre-op 14.31 ± 1.33 to post-op 9.00 ± 1.33 (*p* = 0.0120)) and Widmer (data only shown graphically) showed significant reduction across their study cohorts. Kläusler [8] reports improvement in only diplegic patients across both their post-operative reviews, while Tsang [7] showed no significant improvement in both their groups.

**Fig. 4:**
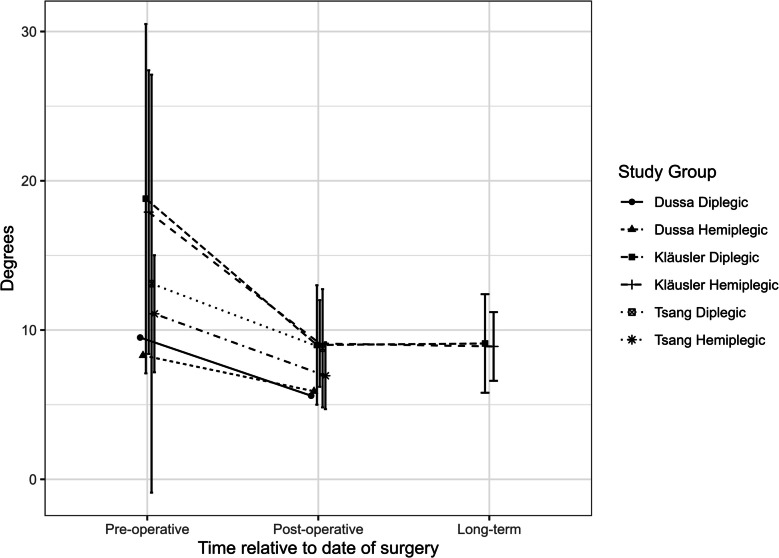
Summary of GPS [24] data from included studies. Error bars provided as standard deviation where available from original studies; pre-operative follow-up time point not specified for any of the included studies; Dussa [10] mean duration of post-operative follow-up = 19.7 months; Kläusler [8] mean duration of post-operative follow-up = 1.3 years; Kläusler mean duration of long-term follow-up = 5.8 years; Tsang [7] mean duration of post-operative follow-up = 17.1 months

Figure 4 shows a graphical summary of GPS data from the included studies.

### Gait Variable Score (GVS) ankle

All studies across all groups showed a decrease in ankle GVS. Subjects were represented by a single centre [6, 8, 9], had the highest pre-operative GVS, but also the greatest improvement post-operatively. Children reported by Dussa and Tsang had less severe pre-operative gait pathology and showed less improvement. Kläusler was the only study to utilise long-term follow-up and showed that improvements persisted at follow-up greater than 5 years.

**Fig. 5:**
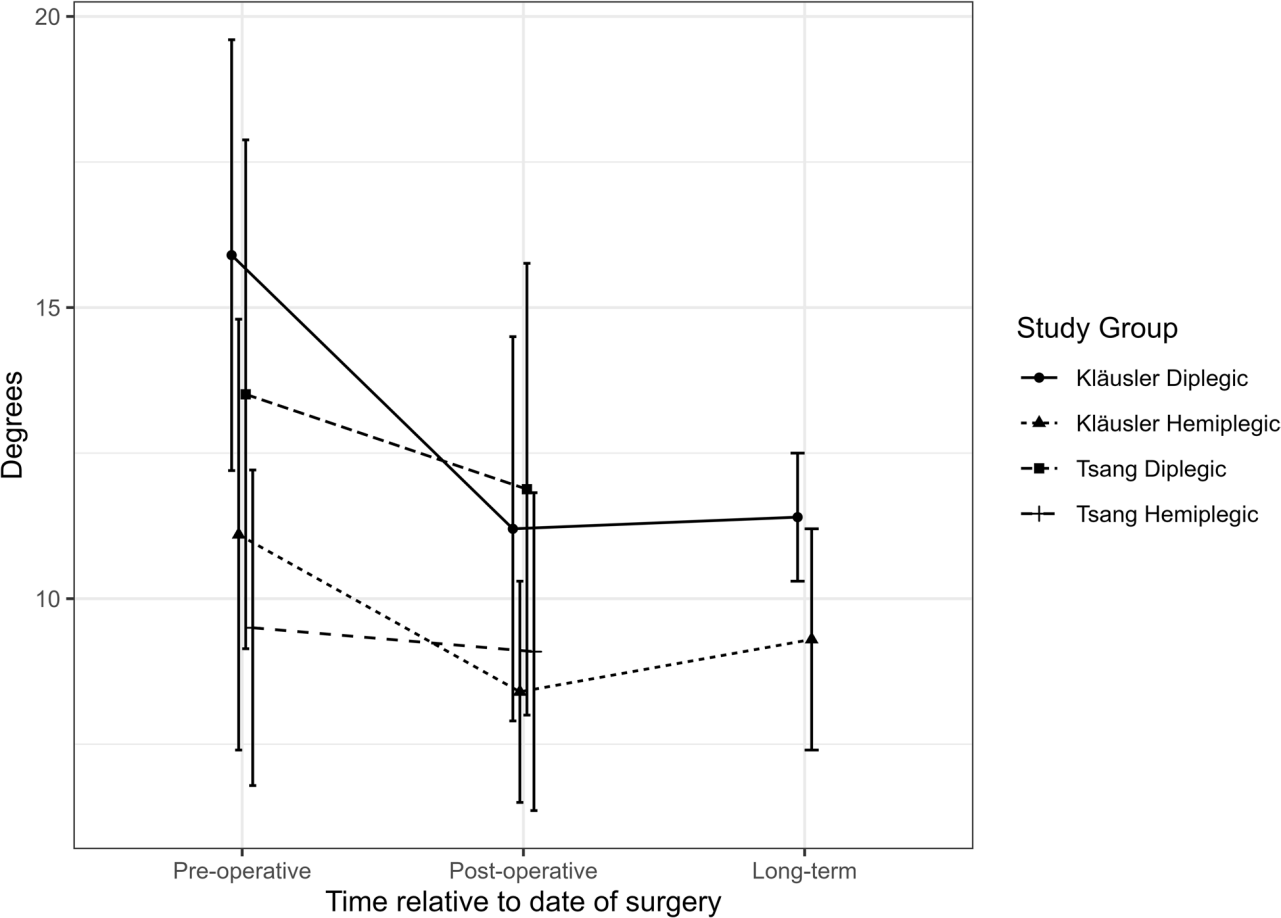
Summary of GVS [24] data across included studies. Error bars provided as standard deviation where available from original studies; pre-operative follow-up time point not specified for any of the included studies; Kläusler’s mean duration of post-operative follow-up = 1.3 years; Kläusler’s mean duration of long-term follow-up = 5.8 years; Tsang’s duration of post-operative follow-up = 17.1 months

Figure 5 shows a graphical summary of GVS data from the included studies.

### AFO use

Widmer [9] reported a reduction in AFO use from 17 of their 22 subjects pre-operatively to three subjects post-operatively. Kläusler [8] showed all subjects required an AFO pre-operatively; however, none were required post-operatively. Five of 28 participants in the study of Tsang [7] ’sstudy no longer required AFO; 14 had easier fitting, and the remainder had no change.

### Complications

Kläusler [8] found three subjects who had relapsed between first and second follow-up, which were hence excluded from the cohort as they eventually required further orthopaedic surgery for recurrent equinus. Besides this, Tsang [7] reported one case of sural nerve neuropraxia which had resolved by the time of post-operative gait analysis. This was more likely to be related to the GSL procedure given the sural nerve’s location in the posterior calf [25]. Therefore, a total of four grade II complications were recorded as per the modified Clavien-Dindo classification system [26, 27].

Across the five papers, there was significant heterogeneity in data reporting. Rutz [6] and Dussa [10] both studied temporospatial parameters, finding no significant change post-operatively. Tsang [7] and Dussa [10] reported on the kinematics of the ankle; however, this was at different points in the gait cycle, thus prohibiting comparison. Rutz [6] found a significant increase in muscle strength; however, this was not maintained at long-term follow-up, as reported by Kläusler [8]. Measurement of manual muscle strength also varied, with Rutz [6] and Kläusler [8] assessing individual muscles, gastrocsoleus and tibialis anterior, while Dussa [10] and Widmer [9] assessed motion such as dorsiflexion and plantarflexion. Tsang [7] published a diagram detailing participant’s pre and post-operative ankle rockers, showing pre-operatively that 25 subjects had toe contact and three had flat foot. This improved post-operatively to having 6 toe contacts, 10 flat feet, and 12 heel strikes. Widmer [9] reported that no subjects had a first rocker present pre-operatively; however, three subjects had a first ankle rocker post-operatively. Further detail of clinical data reported across the studies can be found in the supplementary file.

## Discussion

### Summary of findings

The results of these studies suggest that TATS is a safe addition to the GSL procedure, with a low rate of complications and apparent benefits in terms of improvements in gait kinematics. This is supported by a reduction in post-operative GVS, ankle GPS findings, and reduced AFO use when compared to pre-operative assessment.

The main challenge in synthesising the evidence was the nature of the included studies, comprising case series with small sample sizes. Prior published reviews were utilised to guide the synthesis of evidence and preparation of the manuscript [11–15].

### Aims of management of equinus in CwCP

The aims of the management of equinus in cerebral palsy are prevention/correction of deformity, promotion of a base of support, facilitation of training skills, and an improvement in gait efficiency [2]. While GSL does achieve a number of these goals, it remains to be seen whether the addition of TATS could lead to a greater improvement.

To achieve this gain of function, the surgical procedure would need to both allow the ankle to dorsiflex in the swing phase of gait and plantar flex during the push-off phase of gait. In a subject with spastic cerebral palsy, this would involve lengthening gastrocsoleus to reduce its spasticity while mechanical stretching allows for the foot to be in a greater degree of dorsiflexion [7]. Theoretically, the addition of TATS would allow for greater engagement of this muscle to assist with active dorsiflexion and mechanically for the foot to remain in a more dorsiflexed position [6], thus reducing the chance of post-operative foot drop.

### Differences between unilateral and bilateral cerebral palsy

Sclavos [5] studied post-GSL foot drop in CwCP. They assessed tibialis anterior selective motor control (SMC) using the Boyd and Graham [28] classification. It was seen that both pre-operative ankle dorsiflexor weakness and impaired SMC were predictors for post-operative foot drop in children with hemiplegia. Conversely, these factors showed improvement after surgery in children with diplegia. These differences were attributed to fundamental lesional differences between hemiplegic and diplegic cerebral palsy.

### Proposed explanations for GSL in combination with TATS efficacy

There are two possible mechanisms through which TATS may contribute to an enhanced gait. Bland [29] studied tibialis anterior muscle architecture, strength, and gait in individuals with cerebral palsy using ultrasound imaging, strength testing, and gait analysis. They found that the reduction in muscle strength is partly explained by muscle thickness and cross-sectional area and suggested that treatments to increase size and strength may contribute to gait improvement. Tsang [7] therefore postulated that equinus deformity in cerebral palsy may be primarily due to antagonist (tibialis anterior) weakness rather than agonist (gastrocsoleus) spasticity and therefore suggested GSL in combination with TATS for equinus deformity is analogous to hamstring lengthening and patellar tendon shortening for flexed knee deformity [30].

Nevertheless, while increased tibialis anterior strength would be the optimal outcome, Sclavos [31] suggested in their review that the improved dorsiflexion during the swing phase was a result of mechanical shortening of the tibialis anterior. This meant the ankle was held in dorsiflexion without increasing muscle strength or activation. This is supported by data from Kläusler [8] and Dussa [10], which tested muscle strength pre and post-operatively and found no significant change.

### Predicting outcomes pre-operatively

Lofterød [32] investigated if persistent foot drop could be predicted pre-operatively in children with unilateral cerebral palsy undergoing GSL for equinus deformity. They divided subjects into 5 types based on tibialis anterior muscle activation, with 1 being normal and 5 being paralytic.

Dussa [10] suggested a similar classification can be applied to subjects with bilateral cerebral palsy. They postulated that subjects with good tibialis anterior activity pre-operatively would have minimal benefit from TATS, as the muscle would have sufficient function to dorsiflex the foot and avoid foot drop. Alternatively, subjects with weak tibialis anterior activity may benefit from TATS; however, this was not conclusively proven in their study. Conversely, Tsang [7] suggested that participants with poor selective control of tibialis anterior should not undergo TATS, as they are highly likely to remain AFO-reliant; however, participants with good tibialis anterior control are likely to experience good outcomes.

## Limitations

The principal limitation of this systematic review relates to the small number of included studies and the methodological weakness of the studies. There was no control group or stratification by hemiplegia/diplegia, GMFCS, or SMC. Nevertheless, this is not uncommon following the introduction of a novel surgical technique, as early studies usually concentrate on surgical technique and safety, as seen with previous publications leading to the popularisation of femoral derotation osteotomy for femoral anteversion in CwCP [33, 34]. Additionally, when GSL in combination with TATS was performed as a part of single event multilevel surgery [35–37], the post-operative gait analysis data may suffer a degree of confounding. Furthermore, this review focused on only one procedure for the treatment of equinus deformity and therefore does not compare its effectiveness to other treatments. Three of the included studies were completed at a single institution and studied a similar population over comparable timepoints. To account for sample dependence, out of the three studies completed at the same institution, this review focused on Kläusler [8]. This paper included both unilateral and bilateral procedures and included long-term follow-up and was therefore the best representation of all three studies.

## Future directions

Future studies, ideally in the form of a randomised control trial (RCT), need to focus on stratifying subjects to identify indications for the procedure. Recording pre-/post-operative SMC [28] of tibialis anterior activity would allow researchers to better stratify subjects and predict outcomes. Inclusion of pre-/post-operative 3D ultrasound of tibialis anterior muscle would assist in identifying if tibialis anterior muscle strength or morphology changed as a result of the TATS. Patient-reported outcome measures (PROM) specifically related to gait may assist with assessing patient satisfaction with the procedure. All outcomes should be recorded in a standardised manner, such as using similar 3D camera-based systems for measurement of gait scores, force plates for assessment of power, and surface EMG to quantify muscle activity. This would allow for comparison of data across studies. Most importantly, future studies must include a prospective comparison between GSL only and the GSL in combination with the TATS procedure to quantify the effect of TATS on post-operative outcomes.

## Conclusions

GSL in combination with TATS appears to be safe for the management of equinus gait and equinus deformity in CwCP, demonstrating a low rate of complications. The GSL in combination with TATS procedure appears to lead to improvements in post-operative GVS ankle, GPS findings, and reduced AFO use while offering theoretical advantages for swing phase gait kinematics and avoidance of foot drop. However, there is no comparative data to date to support this contention, and studies with a stronger study design are required.

## Supplementary Information

Supplementary Material 1.

## Data Availability

The full search strategy, and all data extracted from included records is included in the supplementary file.
