# Supplementary material for: Gastrocsoleus lengthening in combination with tibialis anterior tendon shortening for equinus deformity in children with cerebral palsy: a systematic review
Source: Syst Rev. 2025 Nov 20;14:232. doi: 10.1186/s13643-025-02985-y (PMC12636153; doi:10.1186/s13643-025-02985-y)
Supplement: Supplementary file 1 — Supplementary Material 1. [file 13643_2025_2985_MOESM1_ESM.docx]

# SUPPLEMENTARY FILE

Gastrocsoleus lengthening in combination with tibialis anterior tendon shortening for equinus deformity in children with cerebral palsy: a systematic review

Contents

[SEARCH STRATEGIES 2](#_Toc186538148)

[MINORS CRITERIA 4](#_Toc186538156)

[DATA COLLECTION 5](#_Toc186538157)

### SEARCH STRATEGIES

#### Embase (Search completed 6^th^ July 2024)

| # | Term | Results |
| --- | --- | --- |
| 1. | (cerebral-pals* or spastic-diplegi* or spastic-hemiplegi* or little-disease or little's-disease or littles'-disease or littles-disease or brain-pals* or brain-paralys* or central-paralys* or cerebral-paralys* or cerebral-pares* or encephalopathia-infantilis or spastic-quadriplegi* or central-pals* or diplegia-spastica).tw,kf,dq. | 46538 |
| 2. | Cerebral palsy/ | 50998 |
| 3. | 1 or 2 | 57538 |
| 4. | pes equinus/ | 1991 |
| 5. | (equinus-deformit* or equinus-contracture* or Talipes-equinu* or Talipes-equino-valgus or Talipes-equinovalgus or Pes-equinus or Equinus-foot).tw,kf,dq. | 1199 |
| 6. | 4 or 5 | 2423 |
| 7. | exp tendon surgery/ | 20008 |
| 8. | exp tendon/ | 59807 |
| 9. | exp ankle joint/ | 4461 |
| 10. | (Reduction or correction* or Operat* or Reconstruct* or repair* or Surger* or Surgical* or Surgeon* or Lengthen* or Tenotom* or Shorten* or tibialis-anterior or gastrocnemius or soleus or gastrocsoleus).tw,kf,dq. or su.fs. | 8486991 |
| 11. | 7 or 8 or 9 or 10 | 8515804 |
| 12. | 3 and 6 and 1 | 459 |

#### Medline (Search completed 6^th^ July 2024)

| # | Term | Results |
| --- | --- | --- |
| 1. | (cerebral-pals* or spastic-diplegi* or spastic-hemiplegi* or little-disease or little's-disease or littles'-disease or littles-disease or brain-pals* or brain-paralys* or central-paralys* or cerebral-paralys* or cerebral-pares* or encephalopathia-infantilis or spastic-quadriplegi* or central-pals* or diplegia-spastica).tw,kf. | 30516 |
| 2. | Cerebral palsy/ | 24471 |
| 3. | 1 or 2 | 34489 |
| 4. | Equinus Deformity/ | 475 |
| 5. | (equinus-deformit* or equinus-contracture* or Talipes-equinu* or Talipes-equino-valgus or Talipes-equinovalgus or Pes-equinus or Equinus-foot).tw,kf. | 766 |
| 6. | 4 or 5 | 987 |
| 7. | Tenotomy/ or Tendons/ or Ankle joint/ | 50406 |
| 8. | (Reduction or correction* or Operat* or Reconstruct* or repair* or Surger* or Surgical* or Surgeon* or Lengthen* or Tenotom* or Shorten* or tibialis-anterior or gastrocnemius or soleus or gastrocsoleus).tw,kf. or su.fs. | 6300118 |
| 9. | 7 or 8 | 6320628 |
| 10. | 3 and 6 and 9 | 219 |

#### Google Scholar (Search completed 6^th^ July 2024)

### "Cerebral Palsy" equinus Tibialis anterior Surgery|procedure

### The first 200 search results were retrieved from this search

#### Web of Science (Search completed 6^th^ July 2024)

| # | Term |
| --- | --- |
| #1 | title/abstract  “cerebral-pals*” OR “spastic-diplegi*” OR “spastic-hemiplegi*” OR “little-disease” OR “little's-disease” OR “littles'-disease” OR “littles-disease” OR “brain-pals*” OR “brain-paralys*” OR “central-paralys*” OR “cerebral-paralys*” OR “cerebral-pares*” OR “encephalopathia-infantilis” OR “spastic-quadriplegi*” OR “central-pals*” OR “diplegia-spastica” |
| #2 | title/abstract  “equinus-deformit*” OR “equinus-contracture*” OR “Talipes-equinu*” OR “Talipes-equino-valgus “ OR “Talipes-equinovalgus” OR “Pes-equinus” OR “Equinus-foot” |
| #3 | title/abstract  “Reduction” OR “correction*” OR “Operat*” OR “Reconstruct*” OR “repair*” OR “Surger*” OR “Surgical*” OR “Surgeon*” OR “Lengthen*” OR “Tenotom*” OR “Shorten*” OR “tibialis-anterior” OR “gastrocnemius” OR “soleus” OR “gastrocsoleus” |
| #4 | #1 AND #2 AND #3 AND #4 |

### Total of 148 results were retrieved from this search

### MINORS CRITERIA

| Risk of Bias Analysis | Rutz 2011 | Tsang 2016 | Kläusler 2017 | Dussa 2021 | Widmer 2024 |
| --- | --- | --- | --- | --- | --- |
| Clearly Stated Aim | 2 | 2 | 2 | 2 | 2 |
| Inclusion of consecutive patients | 1 | 1 | 0 | 1 | 1 |
| Prospective collection of data | 0 | 2 | 0 | 0 | 0 |
| Endpoints appropriate to the aim of the study | 2 | 2 | 2 | 2 | 2 |
| Unbiased assessment of study endpoint | 2 | 2 | 2 | 1 | 2 |
| Follow-up period appropriate to aim of study | 2 | 2 | 2 | 2 | 2 |
| Loss to follow-up <5% | 0 | 0 | 0 | 0 | 0 |
| Prospective calculation of study size | 0 | 1 | 0 | 0 | 0 |
| Total/16 | **9** | **12** | **8** | **8** | **9** |
|  |  |  |  |  |  |
| Additional criteria for comparative studies |  |  |  |  |  |
| An adequate control group | NA | NA | NA | NA | NA |
| Contemporary groups | NA | NA | NA | NA | NA |
| Baseline equivalence of groups | NA | NA | NA | NA | NA |
| Adequate statistical analysis | NA | NA | NA | NA | NA |
| Total/24 | NA | NA | NA | NA | NA |

### DATA COLLECTION

|  | **Study** | **Rutz 2011** | **Tsang 2016** | **Klausler 2017** | **Dussa 2021** | **Widmer 2024** |
| --- | --- | --- | --- | --- | --- | --- |
|  | Study design | Retrospectve Cohort | Prospectve Cohort | Retrospectve Cohort | Retrospectve Case Control Study | Retrospectve Cohort |
|  | Journal and IF | Gait Posture (IF: 2.716 - Scopus) | Gait Posture (IF: 2.716 - Scopus) | Gait Posture (IF: 2.716 - Scopus) | Gait Posture (IF: 2.716 - Scopus) | JCO (IF: 1.854 - Scopus) |
|  | Level of evidence |  |  |  |  |  |
|  | **Conclusion** | We conclude that TATS in combinaton | The present study has independently | TATS in combinaton with TAL | To conclude, the recovery of the DE | Tibialis anterior tendon shortening and |
|  |  | with TAL in spastc equinus in CP is a | confrmed favourable outcomes of | shows a satsfactory long-term | thereby the positon of the foot and | tendon Achilles lengthening |
|  |  | safe procedure and improves but not | combined TATS with CMTL. It has | result afer 5.8 years in the | the ROM of DESwing are primarily due | combinaton yielded positve |
|  |  | completely corrects foot positoning | been found to be reproducible in | correcton of fxed equinus and | to the release of the ‘tether efect’ of | outcomes, showing increased foot |
|  |  | during gait. For the treatment of | terms of safety and efcacy. TATS | drop foot in children with CP. | the calf muscle. However the credit of | dorsifexion, frst ankle rocker |
|  |  | spastc equinus in CP we recommend | combined with CMTL is a | Postoperatvely all subjects were | this statement goes to Davids who | presence, and overall improved gait |
|  |  | shortening of the elongated antagonist | recommended opton for spastc | able to walk without an AFO. | suspected this efect as a cause for the | quality. These fndings support the |
|  |  | (TATS) in combinaton with lengthening | equinus in ambulatory patents with |  | equinus gait [9]. We found in our study | efectveness of this surgical approach |
|  |  | of the short agonist (TAL) for achieving | CP but patents should have actve |  | group, the TATS did not add much to | for treatng pes equinus in children |
|  |  | optmal postoperatve functon. | dorsifexion pre- operatvely. |  | the improvement of dorsifexion of | with unilateral spastc cerebral palsy. |
|  |  |  |  |  | ankle in those patents showing a swing |  |
|  |  |  |  |  | phase dorsifexion actvity during gait. |  |
|  |  |  |  |  | The role of this surgery when TA is |  |
|  |  |  |  |  | paralytc is questonable as they may |  |
|  |  |  |  |  | remain as non-responders. |  |
|  | **Description of cohort** |  |  |  |  |  |
|  | Total operated limbs | 30 (29 patients) | 28 (26 patients) | 22 (20 patients) | 21 limbs (21 patients) | 22 limbs (22 patents) |
|  | Age (at start of study) |  |  | Average age: 14.9 ± 4.0 years |  |  |
|  |  |  |  | Group I: 13.3 ± 3.0 years | Group 1: 13.5 (6.0-19.0) |  |
|  |  | 15.1 +/- 6.3 (Range 7.0-37.5) | 16.8 (SD +/-5.9, range 10.3-34.5) | Group II: 16.6 ± 5.1 years | Group 2: 10.0 [5.0,34.0] | 13.3 years (+/- 3 years) |
|  | Type of CP (unilateral | Group 1: Hemiplegic CP - 21 |  | Group 1: Spastc hemiplegia - 12 | Group 1: Unilateral CP - 12 | All Unilateral |
|  | (hemi/di) vs bilateral) | Group 2: 1 Bilaterally afected, 3 |  | (unilat operatons) | Group 2: Bilateral CP - 9 |  |
|  |  | Quadriplegic | Group 1: Hemiplegic CP - 13 | Group 2: Spastc diplegia - 8 (6 |  |  |
|  |  |  | Group 2: Diplegic CP - 13 | unilat operatons, 2 bilat) |  |  |
|  | GMFCS (I/II/III) |  | I: 18 II: 8 | I: 8 II: 12 | GMFCS I and II | I:18II4 |
|  |  |  |  | Group 1= I: 7 II: 12 |  |  |
|  |  |  |  | Group 2= I: 1 II: 7 |  |  |
|  | Motor type disorder |  |  |  |  |  |
|  | (spastc, hypertonic, |  |  |  |  |  |
|  | dystonic, mixed) |  |  |  |  |  |
|  | Functonal assessment |  |  |  |  |  |
|  | (gross motor functon |  |  |  |  |  |
|  | measurement), |  |  |  |  |  |
|  | functonal mobility scale |  |  |  |  |  |
|  | **Surgery** |  |  |  |  |  |
|  | SEMLS (min 4) vs single |  | Asynchronus multlevel surgery in 19 | 14 children TATS/GSL only 5 | Many had SEMLS (33/44) |  |
|  | Type of surgery - TATS | TATS was performed distally with | The tbialis anterior tendon was | As described by Rutz et al | The need for TATS was randomly | TATS was performed distally with |
|  | method | transosseous fxaton to the medial | exposed distal to the extensor |  | decided by the surgeon without any | transosseous fxaton to the medial |
|  |  | cuneiform bone (suture with 6 Vicryl1, | retnaculum and freed of paratenon |  | pre-set criteria. The TATS was done | cuneiform bone (suture with 6 Vicryl, |
|  |  | Ethicon Inc., Johnson and Johnson) at | (Fig.1). The ankle was placed into no |  | following CMLP and afer closure of all | Ethicon Inc., Johnson and Johnson untl |
|  |  | the original inserton. The tendon was | more than 10 of dorsifexion. The |  | other wounds to prevent slackening in | 2020, since 2020 use of 2.0 FiberWire, |
|  |  | spanned as much as possible, so that | redundant tendon was then rolled |  | the tendon with the foot held in | Arthrex Manufacturing Inc.) at the |
|  |  | the foot held itself in a plantgrade | around a pair of Gillies forceps to |  | maximum DE. The TATS was done at its | original inserton. The tendon was put |
|  |  | positon (Fig. 1). All surgical procedures | maximum tension and in such a way |  | inserton by double reefng of the | under tension in a way that the foot |
|  |  | were performed by the frst and last | that the rolled-up excess tendon lay |  | tendon and anchoring to the bone | maintained a neutral positon at the |
|  |  | authors. | deep in the wound. The tension in |  | using a non-absorbable monoflament | ankle. |
|  |  |  | the shortened tbialis anterior tendon |  | 1.0 Polydioxanone suture (PDS |  |
|  |  |  | was maintained by the assistant |  | Ethicon®) with the foot held in 90◦. The |  |
|  |  |  | whilst a grasping whip sttch (using a |  | positon of the foot was held at 90◦ |  |
|  |  |  | pre-tensioned No. 6 Vicryl suture |  | untl wound closure and Plaster of |  |
|  |  |  | (Ethicon Inc., Johnson and Johnson)) |  | Pariscast applicaton. Subsequent casts |  |
|  |  |  | was placed into the tendon. The |  | were applied by the operatng surgeon |  |
|  |  |  | suture was then anchored to the |  | with the foot held in 90◦ to prevent |  |
|  |  |  | underlying navicular and medial |  | plantarfexion (PF). |  |
|  |  |  | cuneiform bones at the original |  |  |  |
|  |  |  | inserton of tbialis anterior (Fig. 2). |  |  |  |
|  | Type of surgery - GSL | In all others TAL was performed using | Posterior calf muscle lengthening was | As described by Rutz et al | The type of CMLP performed was | TAL was performed using an open |
|  | method | an open Z lengthening of the Achilles | performed by recessing the proximal |  | determined by the Silfverskj¨old test | Z-lengthening of the Achilles tendon to |
|  |  | tendon with the tendon repaired under | gastrocnemius and, if required, |  | [23]. Those who had a positve test | be able to control the amount of |
|  |  | maximal tension using an absorbable | soleus aponeuroses. |  | received aponeurotc lengthening of | tendon lengthening. The tendon then |
|  |  | suture (6 Vicryl1, Ethicon Inc., Johnson |  |  | the gastrocnemius muscle and those | was repaired under maximal tension |
|  |  | and Johnson) and the foot in 108 of |  |  | with a negatve test, a Strayer [25] or a | using an absorbable suture (6 Vicryl, |
|  |  | dorsifexion in patents with hemiplegia |  |  | Z- lengthening of the Achilles tendon | Ethicon Inc., Johnson and Johnson) |
|  |  | and 5–108 of plantarfexion in diplegia |  |  | was performed depending on the | with the foot in 10° of dorsifexion in |
|  |  | or quadriplegia. |  |  | degree of fxed equinus contracture. | knee extension.9 |
|  |  |  |  |  | The end point of lengthening was a 5◦ |  |
|  |  |  |  |  | passive DE of the ankle joint with |  |
|  |  |  |  |  | extended knee. |  |
|  | Bilateral vs Unilateral |  |  |  |  |  |
|  | Complicatons using |  |  |  |  |  |
|  | Surgical complicaton | Nil complicatons reported | Not mentoned | Not mentoned | Not mentoned | Not mentoned |
|  | Non-surgical |  |  |  |  |  |
|  | Botulinum toxin use | Before TAL a test injecton with | Botulinum toxin in past 6 months was | Botulinum toxin in past 6 months | Botulinum toxin in past 6 months was | Botulinum toxin in past 6 months was |
|  |  | botulinim toxin type A in the | part of exclusion criteria | was part of exclusion criteria. | part of exclusion criteria | part of exclusion criteria |
|  |  | gastro-soleus muscles excluded all |  | Before performing TAL a test |  |  |
|  |  | patents who deteriorated by |  | injecton with botulinum toxin A |  |  |
|  |  | weakening this muscle |  | in the gastro-soleus muscles was |  |  |
|  |  |  |  | performed to exclude all patents |  |  |
|  |  |  |  | who afer the injecton showed a |  |  |
|  |  |  |  | deterioraton by weakening this |  |  |
|  |  |  |  | muscle [18]. |  |  |
|  | **Rehab** |  |  |  |  |  |
|  | Do they describe it | Afer the surgical procedure a castwas | Post-operatvely the foot was placed | Postoperatvely a cast was applied | 6 weeks of Plaster of Paris was | Post-operatvely, a cast was applied in |
|  | (inpatent vs outpatent, | applied in plantgrade (hemiplegia) or | at a plantgrade positon and held in a | in plantgrade (unilateral CP, | followed by an ankle foot orthosis with | plantgrade positon for 6 weeks, with |
|  | use of ankle foot | mild equinus positon (diplegia and | below knee cast which was split | group I) or mild equinus positon | fxed positon of ankle at 90◦ for | weightbearing allowed afer 4 weeks. |
|  | orthoses, sessions per | quardriplegia) for 6 weeks | immediately. Afer two weeks the | (bilateral CP, group II) for 6 weeks | another 6 weeks during walking to | Following cast removal, patents wore a |
|  | week) | postoperatvely, with weight bearing | cast was changed to a plantgrade | postoperatvely. If there was no | facilitate healing and prevent | rigid AFO for 6 weeks, transitoning to a |
|  |  | afer 4 weeks. If actve ankle | weight-bearing cast and retained for | actve ankle dorsifexion afer cast | overlengthening of Tibialis anterior | hinged AFO at 3 months, and gradually |
|  |  | dorsifexion was not possible | a further four weeks. | removal, the child was provided | tendon. Assisted actve plantarfexion | reducing wear-tme based on actve |
|  |  | immediately afer cast removal a |  | with a hinged ankle foot orthosis | and dorsifexion of the ankle was only | ankle dorsifexion during gait. |
|  |  | hinged ankle foot orthosis (AFO) with |  | (AFO) with plantarfexion block. | allowed at 6 weeks following surgery |  |
|  |  | plantarfexion block was fted. |  |  | only during the physiotherapy. A |  |
|  |  |  |  |  | below-knee night-tme bracing with |  |
|  |  |  |  |  | ankle joint at 0◦ was provided during |  |
|  |  |  |  |  | the entre growth. |  |


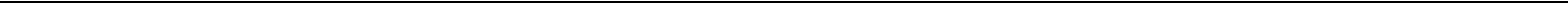

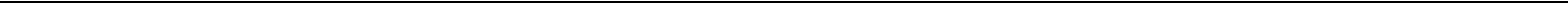

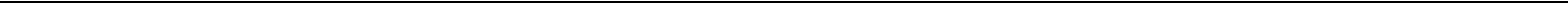

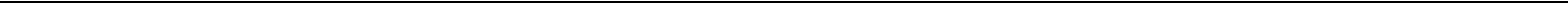

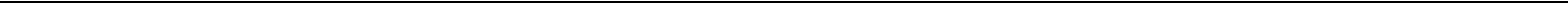


|  | **Study** | **Rutz 2011** | **Tsang 2016** |  | **Klausler 2017** | **Dussa 2021** | **Widmer 2024** |
| --- | --- | --- | --- | --- | --- | --- | --- |
|  | **Assessment (T0)** |  |  |  |  |  |  |
|  | Temporo-spatal | Group 1: |  |  |  | Group 1 |  |
|  | parameter (walking | Walking speed - 1.16 +/- 0.18 |  |  |  | Non-dimensional velocity: 0.41 |  |
|  | speed, stride length, | Cadence - 1.16 +/- 0.18 |  |  |  | [0.32,0.53] |  |
|  | cadence) | Stride length - 1.22 +/- 0.12 |  |  |  | Non-dimensional step length: 66.4 |  |
|  |  | Group 2: 0 |  |  |  | [58.5,89.4] |  |
|  |  | Walking speed - 1.12 +/- 0.06 0 |  |  |  | Non-dimensional cadence: 0.58 |  |
|  |  | Cadence - 2.16 +/- 0.080 |  |  |  | [0.51,0.69] |  |
|  |  | Stride length - 1.06 +/- 0.07 |  |  |  | Group 2 |  |
|  |  |  |  |  |  |  |  |
|  |  |  |  |  |  | Non-dimensional velocity: 0.38 |  |
|  |  |  |  |  |  | [0.34,0.52] |  |
|  |  |  |  |  |  | Non-dimensional step length: 69.6 |  |
|  |  |  |  |  |  | [53.6,89.4] |  |
|  |  |  |  |  |  | Non-dimensional cadence: 0.58 |  |
|  |  |  |  |  |  | [0.55,0.69] |  |
|  | Kinematcs (angles of |  | Group 1 |  |  | Group 1 |  |
|  | gait study) |  | **Maximum ankle dorsifexion during** | |  | Passive knee extension[◦]: 0.0 |  |
|  |  |  | **swing phase 3.41 +/- 12.83** | |  | [-10.0,15.0] |  |
|  |  |  |  |  |  | **Passive ankle dorsifexion knee** |  |
|  |  |  | Group 2 |  |  | **extended [◦]: -7.5 [-20.0,0.0]** |  |
|  |  |  | **Maximum ankle dorsifexion during** | |  | **Passive ankle plantarfexion [◦]: 40.0** |  |
|  |  |  | **swing phase 4.61 +/- 15.27** | |  | **[20.0,70.0]** |  |
|  |  |  |  |  |  | Group 2 |  |
|  |  |  |  |  |  | Passive knee extension[◦]: 0.0 |  |
|  |  |  |  |  |  | [-20.0,5.0] |  |
|  |  |  |  |  |  | **Passive ankle dorsifexion knee** |  |
|  |  |  |  |  |  | **extended [◦]: -10.0** |  |
|  |  |  |  |  |  | **[-20.0,0.0]** |  |
|  |  |  |  |  |  | Passive ankle plantarfexion [◦]: 40.0 |  |
|  |  |  |  |  |  | [20.0,135.0] |  |
|  | Kinetcs (using force |  |  |  |  |  |  |
|  | plates to describe |  |  |  |  |  |  |
|  | forces) |  |  |  |  |  |  |
|  | MSK spastcity | Group 1 |  |  | Group 1 |  |  |
|  |  | **Gastrocnemious: 2.27+/-1.39** |  |  | Gastronemius (range) 1.8 ±1.3 |  |  |
|  |  | **Tibialis anterior: 0.80+/-0.73** |  |  | Tibialis anterior (range) 0.4 ±0.5 |  |  |
|  |  | Group 2 |  |  | Group 2 |  |  |
|  |  | **Gastrocnemious: 1.67+/-1.36** |  |  | Gastronemius (range) 1.9 ±1.1 |  |  |
|  |  | Tibialis anterior: 0.69+/-0.53 |  |  | Tibialis anterior (range) 0.7 ±0.5 |  |  |
|  | Manual muscle strength | Group 1 |  |  | Group 1 | Group 1 | Plantarfexion 3.3 (SD 1.3)0 |
|  |  | Gastrocnemious: 3.46+/-1.06 |  |  | Gastronemius (range) 3.9 ±0.9 | Actve ankle dorsifexion strength: 2.3 | Dorsifexion 3.2 (SD 1.2) |
|  |  | Tibialis anterior: 3.55+/-1.06 |  |  | Tibialis anterior (range) 4.0 ±0.9 | [1.0,4.0] |  |
|  |  | Group 2 |  |  | Group 2 | Group 2 |  |
|  |  | Gastrocnemious: 3.47+/-1.25 |  |  | Gastronemius (range) 3.1 ±1.1 | Actve ankle dorsifexion strength: 3.5 |  |
|  |  | Tibialis anterior: 3.67+/-1.16 |  |  | Tibialis anterior (range) 3.8 ±1.0 | [1.0,4.5] |  |
|  | MSK selectvity |  |  |  |  |  |  |
|  | Gait scores (Melbourne | Group 1 | Group 1 |  | Group I + II |  |  |
|  | gait profle score | **GPS 12.47 +/- 3.80 #** | GPS 9.50 +/- 2.71 |  | **GPS 13.3 ± 4.4** |  |  |
|  | (movement specifc | **GGI 289.51 +/- 239.46 #** |  |  |  |  |  |
|  | profle, GPS) Gait | **GDI 72.05 +/- 9.45 #** | Group 2 |  | Group I |  |  |
|  | deviaton index (USA), |  | GPS 13.51 +/- 4.37 |  | GPS 11.1 ±3.7 |  |  |
|  | gilete gait index) | Group 2 |  |  |  |  |  |
|  |  | **GPS 14.31 +/- 1.33 #** |  |  | Group II |  |  |
|  |  | **GGI 468.133 +/- 97.20 #** |  |  | **GPS 15.9 ±3.7** |  |  |
|  |  | **GDI 65.86 +/- 3.57 #** |  |  |  |  |  |
|  | Clinical assessment |  | Group 1 |  |  |  | Plantarfexion: 26.8 deg (SD 20.8 deg)0 |
|  |  |  | **EVGS operated limb 10.21 +/- 3.07** | |  |  | Dorsifexion Knee extended: -13.9 deg |
|  |  |  | **EVGS non-operated limb 9.71 +/-** | |  |  | (SD 15.0 deg)0 |
|  |  |  | **3.86** |  |  |  | Dorsifexion Knee fexed: -6.1 deg (SD |
|  |  |  | **EVGS total 13.00 +/- 5.87** | |  |  | 12.6 deg) |
|  |  |  | Group 2 |  |  |  |  |
|  |  |  | **EVGS operated limb 12.91 +/- 5.45** | |  |  |  |
|  |  |  | **EVGS non-operated limb 11.00 +/-** | |  |  |  |
|  |  |  | **3.57** |  |  |  |  |
|  |  |  | **EVGS total 24.42 +/- 9.66** |  |  |  |  |
|  | EMG or pedobarography |  |  |  |  |  |  |
|  | Movement analysis | Group 1 | Group 1 |  | Group I + II | Group 1 |  |
|  | profle (MAP) - | MAP pelvic tlt 7.50 +/- 4.65 Ant. to | MAP pelvic tlt 7.46 +/- 7.67 | | MAP pelvic tlt 7.9 ± 3.9 | MAP ankle [◦]: 8.3 [3.5,31.5] |  |
|  | Summerises kinematc | post. | MAP pelvic obliquity 3.52 | +/- 2.50 | MAP pelvic obliquity 3.6 ± 1.5 | Knee extension inital contact [◦]: 20.3 |  |
|  | data from 3D gait | MAP pelvic obliquity 3.94 +/- 2.19 Up | MAP pelvic rotaton 5.27 | +/- 3.36 | MAP pelvic rotaton 9.3 ± 4.2 | [2.5,40.7] |  |
|  | analysis | to down | MAP hip fexion/extension 12.94 +/- | | MAP hip fex/ext 10.6 ±5.6 | Peak knee extension stance [◦]: 9.3 |  |
|  |  | MAP pelvic rotaton 9.16 +/- 5.07 IR to | 7.20 |  | MAP hip abd/add 5.5 ± 2.5 | [-11.7,37.4] |  |
|  |  | ER | MAP hip add/abducton 5.04 +/- | | MAP hip rotaton 15.2 ±10.1 | **Ankle fexion inital contact [◦]: -12.0** |  |
|  |  | MAP hip fex/ext 10.82 +/- 5.18 Flex. to | 2.30 |  | MAP knee fex/ext 15.2 ±8.2 | **[-27.8,-** |  |
|  |  | ext. | MAP hip rotaton 10.00 +/- 7.69 | | **MAP ankle dorsifex/ext 18.3** | **0.3]** |  |
|  |  | **MAP hip abd/add 6.70 +/- 3.02 # Add** | MAP knee fexion/extension 13.81 | | **±10.3** | **Peak ankle dorsifexion [◦]: 8.1** |  |
|  |  | **to abd** | +/- 5.75 |  | MAP foot progression 14.6 ±8.6 | **[-26.7,16.2]** |  |
|  |  | MAP hip rotaton 12.41 +/- 5.93 IR to | **MAP ankle dorsi/plantarfexion** | |  | **Ankle plantarfexion push-of [◦]: -10.4** |  |
|  |  | ER | **11.09 +/- 3.92** |  | Group I | **[-38.9,-** |  |
|  |  | MAP knee fex/ext 11.69 +/- 2.95 Flex. | MAP foot progression 15.00 +/- | | MAP pelvic tlt 6.8 ± 3.1 | **3.1]** |  |
|  |  | to ext. | 7.85 |  | MAP pelvic obliquity 3.3 ± 2.2 | **Peak ankle dorsifexion swing [◦]: - 5.1** |  |
|  |  | **MAP ankle dorsifex/ext 20.64 +/-** |  |  | MAP pelvic rotaton 8.4 ± 5.6 | **[-27.6,2.0]** |  |
|  |  | **11.85 # Plantar to dorsi** | Group 2 |  | MAP hip fex/ext 9.6 ± 6.2 |  |  |
|  |  | MAP foot progression 13.81 +/- 6.41 | MAP pelvic tlt 13.30 +/- 7.20 | | MAP hip abd/add 5.2 ± 3.0 | Group 2 |  |
|  |  |  | MAP pelvic obliquity 3.95 | +/- 1.95 | MAP hip rotaton 11.5 ±6.9 | MAP ankle [◦]: 9.5 [4.7,18.9] |  |
|  |  | Group 2 | **MAP pelvic rotaton 8.59** | **+/- 5.96** | MAP knee fex/ext 12.2 ±7.4 | Knee extension inital contact [◦]: 25.9 |  |
|  |  | MAP pelvic tlt 6.16 +/- 1.35 Post. to | MAP hip fexion/extension 20.74 +/- | | **MAP ankle dorsifex/ext 17.9** | [7.2,53.0] |  |
|  |  | ant. | 9.00 |  | **±9.5** | Peak knee extension stance [◦]: 19.5 |  |
|  |  | MAP pelvic obliquity 3.21 +/- 0.36 Up | MAP hip add/abducton 6.10 +/- | | MAP foot progression 9.8 ± 2.8 | [-5.1,52.5] |  |
|  |  | to down | 3.84 |  |  | Ankle fexion inital contact [◦]: -5.4 |  |
|  |  | MAP pelvic rotaton 7.98 +/- 1.18 IR to | MAP hip rotaton 12.59 +/- 7.95 | | Group II | [-15.7,3.0] |  |
|  |  | ER | MAP knee fexion/extension 17.90 | | MAP pelvic tlt 9.1 ± 4.3 | **Peak ankle dorsifexion [◦]: 3.6** |  |
|  |  | MAP hip fex/ext 13.10 +/- 2.78 Flex. to | +/- 7.72 |  | MAP pelvic obliquity 4.1 ± 2.0 | **[-6.5,16.1]** |  |
|  |  | ext. | MAP ankle dorsi/plantarfexion 13.11 | | MAP pelvic rotaton 10.5 ±3.7 | **Ankle plantarfexion push-of [◦]: -13.6** |  |
|  |  | MAP hip abd/add 6.19 +/- 0.77 Add to | +/- 14.00 |  | MAP hip fex/ext 11.8 ±4.7 | **[-38.9,6.3]** |  |
|  |  | abd | MAP foot progression 13.66 +/- | | MAP hip abd/add 5.9 ± 1.7 | **Peak ankle dorsifexion swing [◦]: -4.5** |  |
|  |  | MAP hip rotaton 14.96 +/- 2.76 IR to | 7.85 |  | MAP hip rotaton 19.8 ±11.8 | **[-15.4,10.7]** |  |
|  |  | ER |  |  | MAP knee fex/ext 18.9 ±8.6 |  |  |
|  |  | MAP knee fex/ext 18.78 +/- 2.82 Flex. |  |  | **MAP ankle dorsifex/ext 18.8** |  |  |
|  |  | to ext. |  |  | **±11.7** |  |  |
|  |  | **MAP ankle dorsifex/ext 22.83 +/- 2.52** |  |  | MAP foot progression 20.5 ±9.6 |  |  |
|  |  | **Plantar to dorsi** |  |  |  |  |  |
|  |  | **MAP foot progression 13.42 +/- 1.81** |  |  |  |  |  |


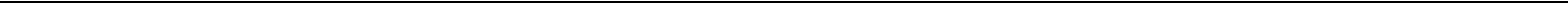


|  | **Study** | | **Rutz 2011** | **Tsang 2016** | |  | **Klausler 2017** | **Dussa 2021** | **Widmer 2024** |
| --- | --- | --- | --- | --- | --- | --- | --- | --- | --- |
|  | **Outcome** | |  |  |  |  |  |  |  |
|  | T1 6-24 months | | Group 1 (Hemiplegic) - 14 months | 17.1 (SD +/- 5.6 months) | |  | 1-2 years (Average 1.3 years) |  |  |
|  |  |  | (9.0-24.0) |  |  |  |  |  |  |
|  |  |  | Group 2 (Bilat/Quadriplegic) - 14.3 |  |  |  |  |  |  |
|  |  |  | months (9.0-24.0) |  |  |  |  |  |  |
|  | Temporo-spatal | | Group 1: |  |  |  |  | Group 1 |  |
|  | parameter (walking | | Walking speed - 1.20 +/- 0.21 |  |  |  |  | Non-dimensional velocity: 0.34 |  |
|  | speed, stride length, | | Cadence - 1.90 +/- 0.19 |  |  |  |  | [0.23,0.44] |  |
|  | cadence) | | Stride length -1.26 +/- 0.15 |  |  |  |  | Non-dimensional step length: 60.6 |  |
|  |  |  |  |  |  |  |  | [50.0,67.3] |  |
|  |  |  | Group 2: |  |  |  |  | Non-dimensional cadence: 0.55 |  |
|  |  |  | Walking speed - 1.17 +/- 0.06 |  |  |  |  | [0.46,0.60] |  |
|  |  |  | Cadence - 2.09 +/- 0.08 |  |  |  |  |  |  |
|  |  |  | Stride length - 1.12 +/- 0.07 |  |  |  |  | Group 2 |  |
|  |  |  |  |  |  |  |  | Non-dimensional velocity: 0.38 |  |
|  |  |  |  |  |  |  |  | [0.34,0.52] |  |
|  |  |  |  |  |  |  |  | Non-dimensional step length: 69.6 |  |
|  |  |  |  |  |  |  |  | [53.6,89.4] |  |
|  |  |  |  |  |  |  |  | Non-dimensional cadence: 0.58 |  |
|  |  |  |  |  |  |  |  | [0.55,0.69] |  |
|  | Kinematcs (angles of | |  | Group 1 |  |  |  | Group 1 |  |
|  | gait study) | |  | **Maximum ankle dorsifexion during** | | |  | Passive knee extension[◦]: 0.0 |  |
|  |  |  |  | **swing phase 14.08 +/- 4.60** | |  |  | [-10.0,15.0] |  |
|  |  |  |  |  |  |  |  | Passive ankle dorsifexion knee |  |
|  |  |  |  | Group 2 |  |  |  | extended [◦]: 5.0 [0.0,10.0] |  |
|  |  |  |  | **Maximum ankle dorsifexion during** | | |  | Passive ankle plantarfexion [◦]: 22.5 |  |
|  |  |  |  | **swing phase 17.23 +/- 8.08** | |  |  | [10.0,50.0] |  |
|  |  |  |  |  |  |  |  | Group 2 |  |
|  |  |  |  |  |  |  |  | Passive knee extension[◦]: 0.0 |  |
|  |  |  |  |  |  |  |  | [-20.0,5.0] |  |
|  |  |  |  |  |  |  |  | **Passive ankle dorsifexion knee** |  |
|  |  |  |  |  |  |  |  | **extended [◦]: -10.0** |  |
|  |  |  |  |  |  |  |  | **[-20.0,0.0]** |  |
|  |  |  |  |  |  |  |  | Passive ankle plantarfexion [◦]: 40.0 |  |
|  |  |  |  |  |  |  |  | [20.0,135.0] |  |
|  | Kinetcs (using force | |  |  |  |  |  |  |  |
|  | plates to describe | |  |  |  |  |  |  |  |
|  | forces) - difcult on | |  |  |  |  |  |  |  |
|  | GMFCS 3 | |  |  |  |  |  |  |  |
|  | MSK spastcity | | Group 1 |  |  |  | Group 1 |  |  |
|  |  |  | **Gastrocnemious: 0.79 +/- 0.66** |  |  |  | **Gastronemius (range) 0.6 ± 0.5** |  |  |
|  |  |  | **Tibialis anterior: 0.29 +/- 0.46** |  |  |  | Tibialis anterior (range) 0.2 ± 0.0 |  |  |
|  |  |  | Group 2 |  |  |  | Group 2 |  |  |
|  |  |  | **Gastrocnemious: 0.53 +/- 0.52** |  |  |  | Gastronemius (range) 1.0 ± 0.7 |  |  |
|  |  |  | Tibialis anterior: 0.39 +/- 0.47 |  |  |  | Tibialis anterior (range) 0.4 ± 0.5 |  |  |
|  | Manual muscle strength | | Group 1 |  |  |  | Group 1 | Group 1 | Plantarfexion 3.3 (SD 1.1) p = 0.9540 |
|  |  |  | Gastrocnemious: 3.83 +/- 1.00 |  |  |  | Gastronemius (range) 3.9 ± 0.8 | Actve ankle dorsifexion strength: 2.5 | Dorsifexion 3.5 (SD 1.3) p = 0.154 |
|  |  |  | Tibialis anterior: 4.10 +/- 0.92 |  |  |  | Tibialis anterior (range) 4.5 ± 0.7 | [0.0,4.0] |  |
|  |  |  | Group 2 |  |  |  | Group 2 | Group 2 |  |
|  |  |  | Gastrocnemious: 4.00 +/- 1.19 |  |  |  | Gastronemius (range) 3.2 ± 1.0 | Actve ankle dorsifexion strength: 3.0 |  |
|  |  |  | Tibialis anterior: 4.36 +/- 0.93 |  |  |  | Tibialis anterior (range) 4.2 ± 0.9 | [2.0,5.0] |  |
|  | MSK selectvity | |  |  |  |  |  |  |  |
|  | Gait scores (Melbourne | | Group 1 | Group 1 |  |  | Group I + II |  |  |
|  | gait profle score | | **GPS 9.18 +/- 2.55 #** | GPS 9.09 | +/- 2.73 |  | **GPS 9.7 ± 3.2** |  |  |
|  | (movement specifc | | **GGI 132.96 +/- 96.24 #** |  |  |  |  |  |  |
|  | profle, GPS) Gait | | **GDI 82.61 +/- 9.24 #** | Group 2 |  |  | Group I |  |  |
|  | deviaton index (USA), | | **Group 2** | GPS 11.88 | +/- 3.88 |  | GPS 8.4 ± 1.9 |  |  |
|  | gilete gait index) | |  |  |  |  |  |  |  |
|  |  |  | **GPS 9.00 +/- 1.33 #** |  |  |  | Group II |  |  |
|  |  |  | **GGI 131.02 +/- 97.20 #** |  |  |  | **GPS 11.2± 3.3** |  |  |
|  |  |  | **GDI 81.31 +/- 3.57 #** |  |  |  |  |  |  |
|  | Clinical assessment | |  | Group 1 |  |  |  |  | Plantarfexion 36.1 deg (SD 14.1 deg) p |
|  |  |  |  | **EVGS operated limb 8.29 +/- 3.91** | | |  |  | = 0.0670 |
|  |  |  |  | **EVGS non-operated limb 2.50** | | **+/-** |  |  | **Dorsifexion Knee extended -1.3 deg** |
|  |  |  |  | **2.94** |  |  |  |  | **(SD 9.6 deg) 0** |
|  |  |  |  | **EVGS total 10.79 +/- 6.55** | |  |  |  | **Dorsifexion Knee fexed 4.8 deg (SD** |
|  |  |  |  | Group 2 |  |  |  |  | **7.1 deg)** |
|  |  |  |  |  |  |  |  |  |  |
|  |  |  |  | **EVGS operated limb 8.86 +/- 5.21** | | |  |  |  |
|  |  |  |  | **EVGS non-operated limb 7.83** | | **+/-** |  |  |  |
|  |  |  |  | **5.98** |  |  |  |  |  |
|  |  |  |  | **EVGS total 18.15 +/- 10.06** | |  |  |  |  |
|  | EMG or pedobarography | |  |  |  |  |  |  |  |

|  | **Study** | | **Rutz 2011** | **Tsang 2016** |  | **Klausler 2017** | **Dussa 2021** | **Widmer 2024** |
| --- | --- | --- | --- | --- | --- | --- | --- | --- |
|  | Movement analysis | | Group 1 | Group 1 |  | Group I + II | Group 1 |  |
|  | profle (MAP) | | MAP pelvic tlt: 6.73 +/- 3.26 Ant. to | MAP pelvic tlt 7.53 +/- 7.29 | | MAP pelvic tlt 8.9 ± 6.5 | MAP ankle [◦]: 5.9 [3.4,11.7] |  |
|  |  |  | post. | MAP pelvic obliquity 3.77 | +/- 2.83 | MAP pelvic obliquity 3.2 ± 1.5 | Knee extension inital contact [◦]: 19.0 |  |
|  |  |  | MAP pelvic obliquity: 3.80 +/- 2.03 Up | MAP pelvic rotaton 5.03 | +/- 1.88 | MAP pelvic rotaton 8.0 ± 3.8 | [14.0,27.0] |  |
|  |  |  | to down | MAP hip fexion/extension 11.43 +/- | | MAP hip fex/ext 10.6± 6.0 | Peak knee extension stance [◦]: 14.4 |  |
|  |  |  | MAP pelvic rotaton: 7.67 +/- 4.04 IR to | 7.29 |  | MAP hip abd/add 5.2 ± 2.2 | [-0.6,26.2] |  |
|  |  |  | ER | MAP hip add/abducton 4.34 +/- | | MAP hip rotaton 9.8 ± 7.3 | Ankle fexion inital contact [◦]: -4.0 |  |
|  |  |  | MAP hip fex/ext: 8.54 +/- 4.44 Flex. to | 2.38 |  | MAP knee fex/ext 11.5±5.4 | [-9.7,6.9] |  |
|  |  |  | ext. | MAP hip rotaton 11.66 +/- 7.84 | | **MAP ankle dorsifex/ext 9.0 ± 3.4** | Peak ankle dorsifexion [◦]: 15.2 |  |
|  |  |  | **MAP hip abd/add: 4.96 +/- 2.21 # Add** | MAP knee fexion/extension 11.89 | | MAP foot progression 10.3± 4.9 | [2.8,24.0] |  |
|  |  |  | **to abd** | +/- 6.00 |  |  | Ankle plantarfexion push-of [◦]: -0.9 |  |
|  |  |  | MAP hip rotaton: 10.95 +/- 6.42 IR to | **MAP ankle dorsi/plantarfexion 6.94** | | Group I | [-14.0,14.8] |  |
|  |  |  | ER | **+/- 2.24** |  | MAP pelvic tlt 6.4 ± 3.5 | Peak ankle dorsifexion swing [◦]: 2.9 |  |
|  |  |  | MAP knee fex/ext: 10.36 +/- 3.17 Flex. | MAP foot progression 10.89 +/- | | MAP pelvic obliquity 3.1 ± 1.6 | [-7.2,14.9] |  |
|  |  |  | to ext. | 5.41 |  | MAP pelvic rotaton 6.7 ± 2.9 |  |  |
|  |  |  | **MAP ankle dorsifex/ext: 9.62 +/- 5.48** |  |  | MAP hip fex/ext 9.4 ± 5.8 | Group 2 |  |
|  |  |  | **# Plantar to dorsi** | Group 2 |  | MAP hip abd/add 5.2 ± 2.7 | MAP ankle [◦]: 5.6 [3.5,8.9] |  |
|  |  |  | MAP foot progression: 10.84 +/- 5.40 | MAP pelvic tlt 14.87 +/- 7.82 | | MAP hip rotaton 9.1 ± 4.3 | Knee extension inital contact [◦]: 17.8 |  |
|  |  |  |  | MAP pelvic obliquity 4.84 | +/- 5.11 | MAP knee fex/ext 9.5 ± 4.6 | [5.5,29.7] |  |
|  |  |  | Group 2 | **MAP pelvic rotaton 5.61 +/- 4.21** | | **MAP ankle dorsifex/ext 9.1 ± 2.9** | Peak knee extension stance [◦]: 15.4 |  |
|  |  |  | MAP pelvic tlt 6.16 +/- 1.35 Post. to | MAP hip fexion/extension 19.88 +/- | | MAP foot progression 8.6 ± 3.2 | [0.7,26.2] |  |
|  |  |  | ant. | 7.60 |  |  | Ankle fexion inital contact [◦]: -5.2 |  |
|  |  |  | MAP pelvic obliquity 3.21 +/- 0.36 Up | MAP hip add/abducton 5.98 +/- | | Group II | [-8.8,10.1] |  |
|  |  |  | to down | 4.62 |  | MAP pelvic tlt 12.0± 7.8 | **Peak ankle dorsifexion [◦]: 11.8** |  |
|  |  |  | MAP pelvic rotaton 7.98 +/- 1.18 IR to | MAP hip rotaton 10.94 +/- 6.91 | | MAP pelvic obliquity 3.4 ± 1.6 | **[4.9,23.3]** |  |
|  |  |  | ER | MAP knee fexion/extension 15.40 | | MAP pelvic rotaton 9.7 ± 4.3 | **Ankle plantarfexion push-of [◦]: - 5.4** |  |
|  |  |  | MAP hip fex/ext 13.10 +/- 2.78 Flex. to | +/- 10.51 |  | MAP hip fex/ext 12.1± 6.2 | **[-18.6,3.9]** |  |
|  |  |  | ext. | MAP ankle dorsi/plantarfexion 8.78 | | MAP hip abd/add 5.1 ± 1.5 | **Peak ankle dorsifexion swing [◦]:** |  |
|  |  |  | MAP hip abd/add 6.19 +/- 0.77 Add to | +/- 3.96 |  | MAP hip rotaton 10.6± 10.0 | **-2.6[-4.8,11.5]** |  |
|  |  |  | abd | MAP foot progression 10.89 +/- | | MAP knee fex/ext 14.0± 5.5 |  |  |
|  |  |  | MAP hip rotaton 14.96 +/- 2.76 IR to | 5.41 |  | **MAP ankle dorsifex/ext 9.0 ± 4.0** |  |  |
|  |  |  | ER |  |  | MAP foot progression 12.4± 5.9 |  |  |
|  |  |  | MAP knee fex/ext 18.78 +/- 2.82 Flex. |  |  |  |  |  |
|  |  |  | to ext. |  |  |  |  |  |
|  |  |  | **MAP ankle dorsifex/ext 7.21 +/- 2.52** |  |  |  |  |  |
|  |  |  | **Plantar to dorsi** |  |  |  |  |  |
|  |  |  | **MAP foot progression 13.42 +/- 1.81** |  |  |  |  |  |
|  | T2 2-7 yrs | |  |  |  | >3 years (Average 5.8 years +/- |  |  |
|  |  |  |  |  |  | 2.1 years) |  |  |
|  | 3 dimensional gait lab | |  |  |  |  |  |  |
|  | Temporo-spatal | |  |  |  |  |  |  |
|  | parameter (walking | |  |  |  |  |  |  |
|  | speed, stride length, | |  |  |  |  |  |  |
|  | cadence) | |  |  |  |  |  |  |
|  | Kinematcs (angles of | |  |  |  |  |  |  |
|  | gait study) | |  |  |  |  |  |  |
|  | Kinetcs (using force | |  |  |  |  |  |  |
|  | plates to describe | |  |  |  |  |  |  |
|  | forces) | |  |  |  |  |  |  |
|  | MSK spastcity | |  |  |  | Group 1 |  |  |
|  |  |  |  |  |  | **Gastronemius (range) 0.7 ±1.1** |  |  |
|  |  |  |  |  |  | Tibialis anterior (range) 0.2 ±0.0 |  |  |
|  |  |  |  |  |  | Group 2 |  |  |
|  |  |  |  |  |  | Spastcity |  |  |
|  |  |  |  |  |  | **Gastronemius (range) 0.8 ±0.8** |  |  |
|  |  |  |  |  |  | **Tibialis anterior (range) 0.1 ±0.3** |  |  |
|  | MSK selectvity | |  |  |  | Group 1 |  |  |
|  |  |  |  |  |  | Gastronemius (range) 3.5 ±1.2 |  |  |
|  |  |  |  |  |  | Tibialis anterior (range) 4.2 ±0.6 |  |  |
|  |  |  |  |  |  | Group 2 |  |  |
|  |  |  |  |  |  | Gastronemius (range) 2.7 ±1.1 |  |  |
|  |  |  |  |  |  | Tibialis anterior (range) 4.0 ±0.7 |  |  |
|  | Gait scores (Melbourne | |  |  |  | Group I + II |  |  |
|  | gait profle score | |  |  |  | **GPS 10.4± 2.7** |  |  |
|  | (movement specifc | |  |  |  |  |  |  |
|  | profle, GPS) Gait | |  |  |  | Group I |  |  |
|  | deviaton index (USA), | |  |  |  | GPS 9.3 ± 1.9 |  |  |
|  | gilete gait index) | |  |  |  | Group II |  |  |
|  |  |  |  |  |  |  |  |  |
|  |  |  |  |  |  | **GPS 11.4± 1.1** |  |  |
|  | Clinical assessment | |  |  |  |  |  |  |
|  | EMG or pedobarography | |  |  |  |  |  |  |
|  | Movement analysis | |  |  |  | Group I + II |  |  |
|  | profle (MAP) | |  |  |  | MAP pelvic tlt 9.3 ± 6.9 |  |  |
|  |  |  |  |  |  | MAP pelvic obliquity 3.3 ± 1.4 |  |  |
|  |  |  |  |  |  | MAP pelvic rotaton 8.4 ± 4.2 |  |  |
|  |  |  |  |  |  | MAP hip fex/ext 10.8± 5.1 |  |  |
|  |  |  |  |  |  | MAP hip abd/add 4.8 ± 1.9 |  |  |
|  |  |  |  |  |  | MAP hip rotaton 13.04 ±6.2 |  |  |
|  |  |  |  |  |  | MAP knee fex/ext 13.0± 6.1 |  |  |
|  |  |  |  |  |  | **MAP ankle dorsifex/ext 8.9 ± 2.7** |  |  |
|  |  |  |  |  |  | MAP foot progression 10.0± 5.2 |  |  |
|  |  |  |  |  |  | Group I |  |  |
|  |  |  |  |  |  | MAP pelvic tlt 7.4 ± 4.1 |  |  |
|  |  |  |  |  |  | MAP pelvic obliquity 3.2 ± 0.9 |  |  |
|  |  |  |  |  |  | MAP pelvic rotaton 6.6 ± 3.5 |  |  |
|  |  |  |  |  |  | MAP hip fex/ext 10.3± 4.9 |  |  |
|  |  |  |  |  |  | MAP hip abd/add 4.0 ± 1.3 |  |  |
|  |  |  |  |  |  | MAP hip rotaton 13.0± 6.4 |  |  |
|  |  |  |  |  |  | MAP knee fex/ext 11.5± 4.3 |  |  |
|  |  |  |  |  |  | **MAP ankle dorsifex/ext 8.9 ± 2.3** |  |  |
|  |  |  |  |  |  | MAP foot progression 8.0 ± 4.0 |  |  |
|  |  |  |  |  |  | Group II |  |  |
|  |  |  |  |  |  | MAP pelvic tlt 10.9± 8.1 |  |  |
|  |  |  |  |  |  | MAP pelvic obliquity 3.6 ± 2.0 |  |  |
|  |  |  |  |  |  | MAP pelvic rotaton 10.4± 4.4 |  |  |
|  |  |  |  |  |  | MAP hip fex/ext 11.6± 5.8 |  |  |
|  |  |  |  |  |  | MAP hip abd/add 5.4 ± 2.0 |  |  |
|  |  |  |  |  |  | MAP hip rotaton 13.3± 6.5 |  |  |
|  |  |  |  |  |  | MAP knee fex/ext 13.8± 7.5 |  |  |
|  |  |  |  |  |  | **MAP ankle dorsifex/ext 9.1 ± 3.3** |  |  |
|  |  |  |  |  |  | MAP foot progression 12.3± 2.0 |  |  |
